# Measuring the shapes of macromolecules – and why it matters

**DOI:** 10.5936/csbj.201309001

**Published:** 2013-12-09

**Authors:** Jie Li, Paul Mach, Patrice Koehl

**Affiliations:** a Genome Center, University of California, Davis, 451 Health Sciences Drive, Davis, CA 95616, United States; b Graduate Group of Applied Mathematics, University of California, Davis, 1, Shields Ave, Davis, CA, 95616, United States; c Department of Computer Science and Genome Center, University of California, Davis, 1, Shields Ave, Davis, CA, 95616, United States

**Keywords:** alpha shape, proteins, solvation energy, ligand binding sites, atomic contacts

## Abstract

The molecular basis of life rests on the activity of biological macromolecules, mostly nucleic acids and proteins. A perhaps surprising finding that crystallized over the last handful of decades is that geometric reasoning plays a major role in our attempt to understand these activities. In this paper, we address this connection between geometry and biology, focusing on methods for measuring and characterizing the shapes of macromolecules. We briefly review existing numerical and analytical approaches that solve these problems. We cover in more details our own work in this field, focusing on the alpha shape theory as it provides a unifying mathematical framework that enable the analytical calculations of the surface area and volume of a macromolecule represented as a union of balls, the detection of pockets and cavities in the molecule, and the quantification of contacts between the atomic balls. We have shown that each of these quantities can be related to physical properties of the molecule under study and ultimately provides insight on its activity. We conclude with a brief description of new challenges for the alpha shape theory in modern structural biology.

## 1. Introduction

The advent of high-throughput technologies and the concurrent advances in information sciences have led to a data revolution in biology. This revolution is most significant in molecular biology, with an increase in the number and scale of “omics” projects over the last decade. Genomics projects for example have produced impressive advances in our knowledge of genes and their encoded protein structures, proteomics initiatives help to decipher the role of post-translation modifications on these structures and provide maps of protein-protein interactions, and functional genomics is the field that attempts to make use of the data produced by these projects to understand protein functions. However, the biggest challenge today is to assimilate this wealth of information into a conceptual framework that will help us decipher life. For example, the current views of the relationship between protein structure and function remain fragmented. We know of their sequences, more and more about their structures, and we have information on their biological activities, but we have difficulties connecting these dots into a knowledgeable whole. We currently lack the experimental and computational tools for directly studying protein structure, function, and dynamics at the molecular and supra-molecular levels. In this paper, we review some of the current developments in building the computational tools that are needed, focusing on the role that geometry plays in these efforts.

It is worth mentioning first that geometric reasoning has been known to play a major role in chemistry and biology for a few decades now. Indeed, molecular structure or shape and chemical reactivity are highly correlated as the latter depends on the positions of the nuclei and electrons within the molecule. Chemists have long used three-dimensional plastic and metal models to understand the many subtle effects of structure on reactivity and have invested in experimentally determining the structure of important molecules. The same applies to biochemistry, where structural genomics projects are based on the premise that the structure of macromolecules implies their function. Physical properties of these molecules are then often expressed in terms of their geometry. For example, potential active sites are often assimilated with cavities [6, 7] while interactions with the environment are quantified through the surface area and/or volume of their shapes [8–12]. This link between solvation and geometry has led to the development of implicit solvent models that play an essential role in improving simulations of molecular dynamics.

Protein dynamics is multi-scale: from the jiggling of atoms (pico-seconds), the domain reorganizations in proteins (micro to milliseconds), protein folding and diffusion (milli-second to seconds), binding and translocation (seconds to minutes). Connecting these different scales is a central problem in polymer physics that remains unsolved, despite numerous theoretical and computational developments (for review, see [13, 14]). Computer simulations play an essential role in all corresponding multi-scale methods, as they provide information at the different scales. Usually, computer simulations of protein dynamics start with a large system containing the protein and many water molecules to mimic physiological conditions. Given a model for the physical interactions between these molecules, their space-time trajectories are computed by numerically solving the equations of motion. These trajectories however are limited in scope. Current computing technologies limit the range of time scales that can be simulated to the microsecond level, for systems that contain up to hundred thousands of atoms [15]. There are many directions that are currently explored to extend these limits, from hardware solutions including the development of specialized computers [16] or by harnessing the power of graphics processor units [17] to the development of simplified models that are computationally tractable and remain physically accurate. Among such models are those that treat the solvent implicitly, reducing the solute–solvent interactions to their mean-field characteristics. These so-called implicit solvent models are often applied to estimate free energy of solute-solvent interactions in structural and chemical processes, folding or conformational transitions of proteins and nucleic acids, association of biological macromolecules with ligands, or transport of drugs across biological membranes [18–27]. The main advantage of these models is that they express solute-solvent interactions as a function of the solute degrees of freedom alone, more specifically on its volume and surface area. In this review, we will discuss how these geometric measures are usually computed for macromolecules.

The paper is organized as follows. The next section provides a brief description of the representations of macromolecules and the mathematical definitions of their boundaries or surfaces. The following section reviews popular methods for computing the geometric measures of macromolecules using their most common representation, i.e. a union of balls. The following section covers our work on the alpha shape theory and its application to measuring macromolecules. The result section provides a small review of recent applications of the alpha shape theory to analyze the structures macromolecules, as well as examples of application for characterizing atomic environments with protein and detecting putative drug target sites in RNA. We then conclude with a discussion of future research directions.

## 2. The geometry of macromolecules

Molecular structure and chemical reactivity are highly correlated as the latter depends on the positions of the nuclei and electrons within the molecule: this correlation is the rationale for high resolution studies of the structures of bio-molecules. Early crystallographers who studied proteins and nucleic acids could not rely—as it is common nowadays—on computers and computer graphics programs for representation and analysis of their structures. They had developed a large array of finely crafted physical models that allowed them to have a feeling for these molecules. These models, usually made out of painted wood, plastic, rubber and/or metal were designed to highlight different properties of the molecule under study. In the space-filling models, such as those of Corey-Pauling-Koltun (CPK) [28, 29], atoms are represented as balls, whose radii are the atoms’ van der Waals radii. The CPK model has now become standard in the field of macromolecular modeling: a bio-molecule is represented as the union of a set of balls, whose centers match with the atomic centers and radii defined by van der Waals parameters. The structure of a biomolecule is then fully defined by the coordinates of these centers, and the radii values. The macromolecular surface is the geometric surface or boundary of these unions of balls. Note that other definitions are possible; this will be discussed in more details below.

### 2.1 Geometric surface of union of balls

**Figure 1:**
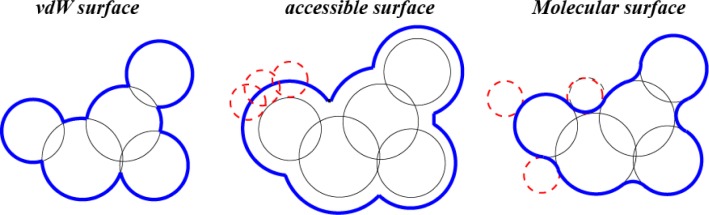
Three molecular surface models (2D examples). Dashed, red circles represent the probe sphere.

As described above, there is no consensus in computational biology as to which surface of the union of balls best relates to the physical properties of the molecule. Three models are widely used; namely, the *van der Waals surface*, the *solvent accessible surface*, and the *molecular surface* (see Figure 1 for a 2D illustration).

The *van der Waals surface*, vdW_B_, is defined as the boundary of the union of balls ∪ *B*. It consists of a number of spherical patches meeting at common circular arcs.

Lee and Richards [8] defined the *solvent accessible surface* *SAS*_*B*_ of a molecule as the locii of the center of a probe sphere with radius *R*_*w*_ as it rolls over the van der Waals surface *vdW*_*B*_. The value of *R*_*w*_ is usually set to 1.4 Å as it approximates the size of a water molecule. It can be shown that *SAS*_*B*_ is also the boundary of the union of balls ∪ *B*_*w*_, where *B*_*w*_ are ”hydrated” balls representing the atoms, i.e. the vdW balls whose radii have been increased by *R*_*w*_.

The *molecular surface*, *MS*_*B*_, was introduced by Richards [10] as an alternate to the van der Waal's surface and the solvent accessible surface. It is defined as the surface traced out by the front of the probe sphere while it rolls over *vdW*_*B*_ (see left panel in Figure 1 for a two dimensional example). The molecular surface consists of three types of patches, namely, spherical patches, toroidal patches and inverse spherical patches.

### 2.2 Alternative representations of macromolecular surfaces

While geometric models (such as the union of balls discussed above) for the molecular surface provide a deterministic description of the boundary for the shape of a macromolecule, surface models using implicit or parametric surfaces may be favorable for certain applications [30, 31].

The implicit molecular surface models use a level set of a scalar function *f:*ℝ^*3*^ *→*ℝ that maps each point from the three dimensional space to a real value [32–34]. The most common scalar function used for macromolecular surfaces is a summation of Gaussian functions [35]. Other scalar functions such as polynomial and Fermi-Dirac switching function have been used as well [36]. Bates et al. [37] proposed the *Minimal Molecular Surface* as a level set of a scalar function that is the output from a numerical minimization procedure.

Parametric surface models specify each point on the macromolecular surface by a pair of real value variables. Piecewise polynomials such as Non-Uniform Rational B-spline (NURBS) and Bernstein-Bézier have been proposed to generate parametric representations for molecular surfaces [30, 38]. Spherical harmonics and their extensions parameterize the macromolecular surface using spherical coordinates and provide a compact analytical representation of macromolecular shapes [39–41].

We note that both implicit and parametric macromolecular surface models are not independent from the geometric models based on union of balls, as they usually have a set of parameters that are tuned such that they provide a reasonable approximation of the surface of the latter. We restrict this section to the description of the macromolecular surface models based on spherical harmonics functions.

Spherical harmonics are single valued complex functions defined on a unit sphere using spherical coordinates *(θ, ϕ)*, that is,

$$Y_{l}^{m}\left(\theta ,\phi \right)=\sqrt{\frac{(2l+1)(l-m)!}{4\pi (l+m)!}}P_{l}^{m}(\cos\theta )\exp(im\phi )$$

in which *l* and *m* are integers with *m* ∈ [-*l, l*] and $P_{l}^{m}(\cos\theta )$ are the associated Legendre polynomials. Any surface *F* that is topologically equivalent to a sphere can be approximated by a linear combination of spherical harmonics basis function

$$F(\theta ,\phi )=\sum_{l=0}^{+\infty }\sum_{m=-l}^{l}C_{lm}Y_{l}^{m}(\theta ,\phi )$$

in which *c*_*lm*_ is the expansion coefficient. Since the spherical harmonics form a complete orthonormal basis, the parameterization of *F*(*θ, ϕ*) is unique and the coefficients are independent [39]. It is possible to build spherical harmonics representations for a macromolecular surface *S* by truncating the infinite series in *l* of the basis functions to a value L that is chosen according to a desired level of approximation. The coefficients *c*_*lm*_ are then evaluated based on a representation of *S* in spherical coordinates [39].

The spherical harmonics representation provides a complete analytical formula for the macromolecular surface. It facilitates multi-resolution approximations of molecular shapes and efficient shape comparison algorithm by taking the expansion coefficients *c*_*lm*_ as shape descriptors [41, 42].

It should be noted that the spherical harmonics representation can only be applied to a macromolecule whose boundary is star like, that is, the radial function *S*(*θ, ϕ*) is single valued. This restriction has limited the application of spherical harmonics based macromolecular surface as many of the macromolecular surfaces have non-zero genera due to the presence of tunnels and overhangs that lead to radial functions *S*(*θ, ϕ*) that are not single valued. To circumvent this problem, an extension of the spherical harmonics called 3D Zernike functions has been proposed for modeling macromolecular surfaces [43–45].

## 3. Measuring macromolecules

A common concrete model representing a molecular shape is a union of balls, in which each ball corresponds to an atom, with its center set at the position of the nucleus of the atom and its radius set to the vdW radius of the atom. In what follows, we discuss the geometric properties of such union of balls, more specifically how we can measure their volume and surface area, how we can detect their pockets and cavities, and how we can quantify interactions between the balls.

### 3.1 Measuring the shape of a macromolecule

Computing the surface area and/or volume of a union of overlapping balls is not a trivial task. The original approach of Lee and Richards [8] computed the surface area by first cutting the union of balls with a set of parallel planes. The intersection of a plane with a ball, if it exists, is a circle that can be partitioned into accessible arcs on the boundary and occluded arcs in the interior of the union. The accessible surface area of an atom *i* is then the sum of the contributions of all its accessible arcs, computed approximately as the product of the arc length and the spacing between the planes defining the arc. This method was originally implemented in the program ACCESS [8]. Shrake and Rupley [46] refined Lee and Richards’ method and proposed a Monte Carlo numerical integration of the accessible surface area. Their method placed 92 points on each atomic sphere, and determined which points were accessible to solvent (not inside any other sphere). Efficient implementations of this method include applications of look-up tables [47], vectorized algorithms [48] and parallel algorithms [49]. Similar numerical methods have been developed for computing the volume of a union of balls [50–53]. It is also worth mentioning MSMS, a program that allows for computing very efficiently an approximation of the surface area of a macromolecule by generating a triangulated version of its surface [54].

The surface area and/or volume computed by numerical integration over a set of points, even if closely spaced, is not accurate and cannot be readily differentiated. To improve upon the numerical methods, analytical approximations to the accessible surface area have been developed, which either treat multiple overlapping balls probabilistically [55–57] or ignore them altogether [58, 59]. While these approaches are approximative, they are fast and lead to differentiable geometric measures. In addition, they are well suited for hardware acceleration on graphics processing units [60].

Even better analytical methods describe the molecule as a union of pieces of balls, each defined by their center, radius, and arcs forming their boundary, and subsequently apply analytical geometry to compute the surface area and volume [61–65]. For example, Pavani and Ranghino [51] proposed a method for computing the volume of a molecule by inclusion-exclusion. In their implementation, only intersections of up to three balls were considered. Petitjean however noticed that practical situations for proteins frequently involve simultaneous overlaps of up to six balls [64]. Subsequently, Pavani and Ranghino's idea was generalized to any number of simultaneous overlaps by Gibson and Scheraga [4] and by Petitjean [64], applying a theorem that states that higher-order overlaps can always be reduced to lower-order overlaps [66]. Doing the reduction correctly remains, however, computationally difficult and expensive. The alpha shape theory solves this problem using Delaunay triangulations and their filtrations, as described by Edelsbrunner [67]. It will be discussed in the next section.

The distinction between approximate and exact computation also applies to existing methods for computing the derivatives of the volume and surface area of a molecule with respect to its atomic coordinates [68–73]. In the case of the derivatives of the surface area, computationally efficient methods were implemented in the MSEED software by Perrot et al. [74] and in the SASAD software by Sridharan et al. [75]. All these methods introduce approximations to deal with singularities caused by numerical errors or by discontinuities in the derivatives [70].

### 3.2 Detecting pockets and cavities in a macromolecule

The problem of detecting and measuring internal cavities of macromolecules is very popular as these cavities correspond to putative binding sites for drugs and thus represent attractive leads for the design of therapeutic drugs. Most solutions to this problem rely heavily on geometry. They can be divided into three categories: (i) the grid-based methods, (ii) the probe sphere detection methods, and (iii) the analytical methods.

In the grid-based method, the molecule is positioned on a three-dimensional Cartesian grid whose vertices are then sorted into two groups: those that are covered by a protein atom and those that are not. The latter are further characterized as being inside a pocket if they satisfy some geometric conditions (such as being inside and at a distance greater than the radius of a water molecule from the convex hull of the macromolecule). The measures of these pockets (volume and surface area) are then computed by Monte Carlo integration over their corresponding grid points. POCKET [76], LIGSITE [77], LigandFit [78], PocketPicker [79], and McVol [53] are cavity-detecting programs that implement this grid-based method.

The probe sphere method proceeds by placing probe spheres that are tangent to the surfaces of two atoms of the biomolecules and then reducing their radii to eliminate overlaps with neighboring atoms; all remaining spheres whose radii exceed a minimal cutoff value (usually 1 Å) are used to define the pockets and cavities. This method was originally implemented in the program SURFNET [80] and later modified in the programs PASS [81] and PHECOM [82]. Interestingly, the grid-based and probe sphere methods were recently combined in the program POCASA [83].

The alpha shape theory combined with the discrete flow concept was the first analytical method proposed for detecting and measuring inaccessible cavities [84] as well as pockets [6, 85] in macromolecules. It has been extended since to detect channels between inner cavities and the outside [86]. The program CAVE implements a complementary approach in which the boundaries of the pockets are directly triangulated, forming the so-called enveloping triangulation [87].

### 3.3 Computing atomic contacts in a macromolecule

While exact theories for computing the surface area and volume of a union of balls exist, the computations of contact areas between balls are more ambiguous as there is no unique definition of what a “contact” is. Three overlapping balls provide a simple illustration of this problem. The regions of the balls that are covered by exactly two balls can be easily partitioned between the corresponding balls. Partitioning the region that is covered by all three balls, however, is more ambiguous. Most methods that compute the contact areas between atoms in a molecule rely on a Voronoi partitioning of such overlapping regions; the contact between two atoms is then defined as the area of the face that separates their Voronoi regions (see for example [88–92]). We note that these methods require special care for atoms on the surface of the molecule of interest, as the corresponding Voronoi cells are unbounded; this is usually resolved by adding water molecules based on molecular dynamics simulations [88, 92]. Finally we mention that Apollonius diagrams (also called additive Voronoi diagrams) have also been used to provide an alternate definition of contacts [93, 94].

## 4. The alpha shape theory: a general framework to characterize the geometry of macromolecules

### 4.1 Volume and surface area of a union of balls

Given a collection *B={B*_*i*_ *}* of *N* three-dimensional balls, the volume and the surface area of the union of *B* can be computed using the principle of *inclusion-exclusion*. That is, the volume and surface area of the union ⋃ *B* can be expressed as an alternating sum of volumes and surface areas of the common intersections of the subsets of *B*,

$$\mu \left(\cup B\right)=\sum_{i=1}^{N}\mu (B_{i})-\sum_{1\leq i<j\leq N}\mu (B_{i}\cap B_{j})+\sum_{1\leq i<j<k\leq N}\mu (B_{i}\cap B_{j}\cap B_{k})-\sum_{1\leq i<j<k<l\leq N}\mu (B_{i}\cap B_{j}\cap B_{k}\cap B_{l})+\cdots ,$$

where *µ* stands for either the volume *V* of the union of balls or the area of its boundary *A*. There are two issues that need to be solved to make this equation computationally tractable. Firstly, we need to have a consistent way to reduce significantly the number of terms in the inclusion-exclusion formula; brute force application would lead to an algorithm with exponential running time, as the total number of terms is *2* ^*N*^ *-1*, with each term corresponding to the measure of the intersection of at most *N* balls. Secondly, we need analytical formula for computing the non-empty intersections of balls.

**Figure 2:**
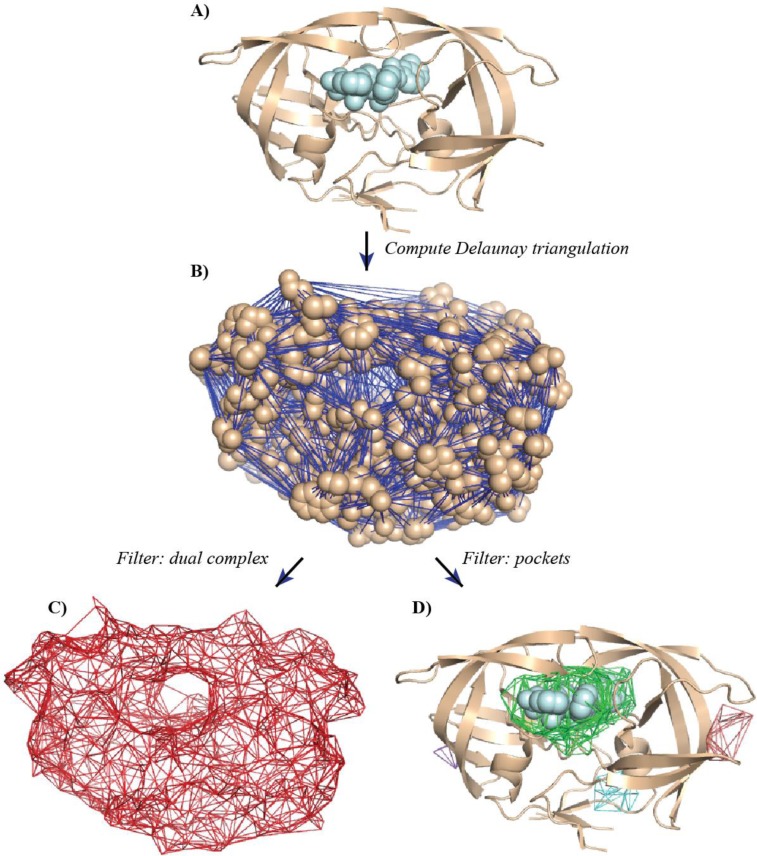
**Measuring the HIV-1 protease using the alpha shape theory**. (A) The structure of the HIV-1 protease (PDB [1] code 3MXE) is shown in cartoon representation. The structure was studied in the presence of an inhibitor, KC32, shown in CPK mode [3]. To compute its geometric properties, we proceed in three steps: (B) first, we compute the weighted Delaunay triangulation (shown as blue edges) of all the atomic balls representing the protein (not including the inhibitor); the Delaunay triangulation is then filtered, to yield the dual complex (C) and a set of pockets (D). The dual complex (in red) is the subset of the Delaunay triangulation that is limited to simplices whose corresponding balls have a non-empty intersection. The largest pocket, shown in green is found to align with the position of KC32 in the protein structure. Three alternate pockets are shown in purple, magenta, and red (D).

The first requirement was elegantly solved with the alpha shape theory. It is based on the concept of Voronoi decompositions and Delaunay triangulations and their filtrations, as proposed by Edelsbrunner [67]. We illustrate its application to measuring the shape of a protein in Figure 2 and describe briefly its major components below. For a more comprehensive description, we refer the reader to the original paper of Edelsbrunner and to some application papers [7, 84, 95, 96]. It is noteworthy however that Naiman and Wynn had introduced the concept of using the Voronoi decomposition and Delaunay triangulation to simplify the inclusion-exclusion formula from a statistical perspective a little earlier [97].

#### Voronoi decomposition and dual complex

Let us consider a finite set of spheres *S*_*i*_ with centers *c*_*i*_ and radii *r*_*i*_ and let *B*_*i*_ be the ball bounded by *S*_*i*_. We define the square distance between a point ***x*** and a sphere *S*_*i*_ as $\pi_{i}\left(X\right)=∥X-c_{i}{∥}^{2}-r_{i}^{2}\cdot$ This distance definition allows for varying radii for the spheres.

The *Voronoi region* *V*_*i*_ of the sphere *S*_*i*_ consists of all points ***x*** that are at least as close to *S*_*i*_ as to any other sphere, *V*_*i*_ *=* {X ∈ ℝ^3^∣*π*_*i*_(X) - *π*_*j*_(X) ∀*j* ≠ *i*}. *V*_*i*_ is a convex polyhedron obtained as the common intersection of finitely many closed half-spaces, one per sphere *S*_*j*_ ≠ *S*_*i*_. The union of all Voronoi regions *V*_*i*_ defines the (weighted) Voronoi diagram, also called the Laguerre diagram of the union of spheres; this union covers the whole space. The intersection of the Voronoi diagram with the union of balls *B*_*i*_ decomposes the union into convex regions of the form *B*_*i*_ ⋂ *V*_*i*_. The boundary of each such region consists of spherical patches on *S*_*i*_ and planar patches on the boundary of *V*_*i*_. The spherical patches separate the inside from the outside and the planar patches decompose the inside of the union.

The weighted Delaunay triangulation is the dual of the weighted Voronoi diagram obtained by drawing an edge between the centers of *S*_*i*_ and *S*_*j*_ if the two corresponding Voronoi regions share a common face, called a Voronoi plane. Furthermore, we draw a triangle connecting ***c***_***i***_, ***c***_***j***_ and ***c***_***k***_ if *V*_*i*_, *V*_*j*_ and *V*_*k*_ intersect in a common line segment, called a Voronoi edge, and similarly we draw a tetrahedron between four centers if their Voronoi regions meet at a common point, called a Voronoi point. Assuming general position of the spheres, there are no other cases to be considered: this is a central property of the Delaunay triangulation that will lead to a significant simplification of the inclusion-exclusion formula (see below).

Let us now limit the construction of the weighted Delaunay triangulation to within the union of balls. In other words, we draw a dual edge between the two vertices ***c***_***i***_ and ***c***_***j***_ only if *B*_*i*_ ⋂ *V*_*i*_ and *B*_*j*_ ⋂ *V*_*j*_ share a common face, and similarly for triangles and tetrahedra. The result is a sub-complex of the Delaunay triangulation, which is referred to as the *dual complex K* of the set of spheres.

It is often useful to alter the spheres by increasing or decreasing their radii (we will see one application in the result section to study pockets in a large RNA molecule). We do this in a way that leaves the Voronoi diagram invariant. Let us model growth with a positive real number denoted *α* ^*2*^. For each *i* let *S*_*i*_(α) be the sphere with center ***c***_***i***_ and radius $r_{i}^{2}+\alpha^{2}$. The *alpha complex K_〈_* of the spheres *S*_*i*_ is the dual complex of the spheres *S*_*i*_(α).

#### Measuring the volume and surface of the union of spheres

As proved in [67], the inclusion-exclusion formula that corresponds to the dual complex gives the correct volume of a union of balls, as well as the correct area of its boundary, the union of spheres. Here we state the corresponding theorem for the volume. Let *s*_*i*_ be the simplex corresponding to the ball *B*_*i*_, *s*_*ij*_ the simplex formed by the edge between the centers of the balls *B*_*i*_ and *B*_*j*_, *s*_*ijk*_ the triangle corresponding to the three balls *B*_*i*_, *B*_*j*_, and *B*_*k*_, and finally *s*_*ijkl*_ the tetrahedron defined by the four balls *B*_*i*_, *B*_*j*_, *B*_*k*_, and *B*_*l*_.

***Volume Theorem:***

$$V\left(\cup B\right)=\sum_{S_{i}}V(B_{i})-\sum_{S_{ij}}\left(V_{i:j}+V_{j:i}\right)+\sum_{S_{ijk}}\left(V_{i:jk}+V_{j:ki}+V_{k:ij}\right)-\sum_{S_{ijkl}}\left(V_{i:jkl}+V_{j:kli}+V_{k:lij}+V_{l:ijk}\right)$$

Here *V(B*_*i*_ *)* is the volume of the ball *B*_*i*_, *V*_*i:j*_ is the contribution of *B*_*i*_ to the volume of the intersection of the balls *B*_*i*_ and *B*_*j*_, etc. A similar theorem is used to compute the surface area *A*. They overcome the exponential complexity of the inclusion-exclusion formula by implicitly reducing higher-order to lower-order overlaps. In addition, we note that the balls in each term form a unique geometric configuration and that the analytic calculations of the volume and surface area can be done without case analysis [67].

Several formulas have been developed for computing the volumes and surface areas of the intersection of two, three and four balls with unequal radii (see for example [4, 98, 99]). Of particular interest to macromolecule structure modeling, we have recently derived new formulas that satisfy a specific constraint, namely that the volume and surface area intersections are only expressed as functions of the radii of the balls and the distances between their centers [96].

#### Detecting pockets in a union of spheres

A full description of how to detect and measure pockets in a union of balls based on the alpha shape theory is available in [6]. Briefly, the concept of pockets is ultimately connected to the notion of a continuous flow field defined on the Delaunay triangulation of these balls. Let *T* be the set of tetrahedra in the Delaunay triangulation and *T' = T* ⋃ *τ*_*∝*_ where *τ*_*∝*_ is a dummy element representing the complement of the triangulation in ℝ^3^. The flow relation '≺' with *τ* ≺ *σ* is defined by:

- *τ* and *σ* share a common triangle ∆, and
- The interior of *τ* and the orthogonal center *Z_τ_* lie on different sides of the plane defined by ∆.

The orthogonal center *Z*_*τ*_ is the center of the smallest ball that is orthogonal to all four balls whose centers are the vertices of *τ*. If *τ* ≺ *σ*, *τ* is said to be a *predecessor* of *σ* and *σ* is then a *successor* of *τ*. *σ* ∈ *T* is a sink if it has no successors; in other words, a tetrahedron is a sink if and only if it contains its orthogonal center. Sinks are important since they are responsible for the formation of voids: if *H* is a void of the union of balls then at least one tetrahedron in *H* is a sink.

By definition, pockets consist of the Delaunay tetrahedra that do not belong to the dual complex *K* and are not ancestors of *τ*_*∝*_. The voids are the only pockets without connection to the outside. All other pockets connect to the outside at one or more places, called *mouth*. Figure 2 illustrates these concepts for the HIV-1 protease. The tetrahedra that form the four major pockets detected by this method are shown overlaid with the structure of the protein. Interestingly, we find that the main pocket (shown in green) matches with the position of the inhibitor detected in the X-ray structure (see Figure 2, panel D) [3].

The surface area and volume of a pocket are easily computed by first identifying their tetrahedra and their faces that belong to the dual complex followed by the application of simplified inclusion-exclusion formulas similar to those used for measuring the dual complex (see [6, 7] for details).

### 4.2 Detecting and measuring contacts between atoms

The computation of contact areas between balls is ambiguous as there is no unique definition of what a contact is [5]. Here we follow the framework of the alpha shape theory described above. The key step when applying this theory to measure a union of spheres is to derive the dual complex *K* of their centers (see above). Two spheres *S*_*i*_ and *S*_*j*_ that are connected by an edge in *K* overlap, i.e. the distance between their centers is smaller than the sum of their respective radii. Based on this observation, we proposed the following definition of contacts between balls [5]:

*Definition:* Two spheres *S*_*i*_ and *S*_*j*_ in a union of spheres ⋃ *S* are said to be in contact if and only if their centers *C*_*i*_ and *C*_*j*_ are connected by an edge in the dual complex *K* of ⋃ *S*.

**Figure 3:**
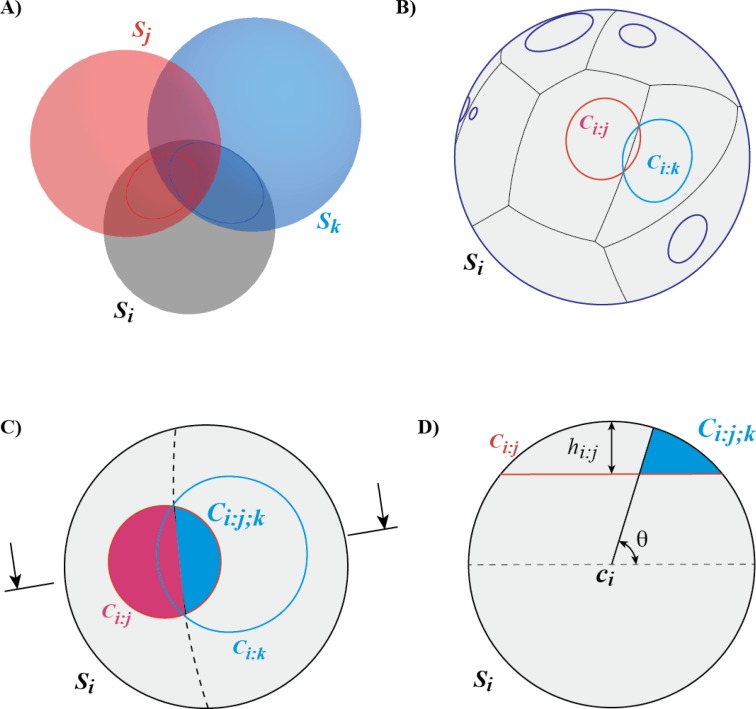
**Computing contact areas between overlapping spheres**. **A)** Let us consider a sphere *S*_*i*_ in contact with two other spheres, *S*_*j*_ and *S*_*k*_, such that the corresponding caps *C*_*i:j*_ and *C*_*i:k*_ overlap. To remove the ambiguity in dividing the overlap area we construct the Laguerre-Voronoi diagram [2] on the surface of the sphere **(B)**. This construction creates Voronoi regions for each cap and separates them with geodesic arcs (here we show the two regions corresponding to the two caps *C*_*i:j*_ and *C*_*i:k*_ in presence of other caps). **C)** The Laguerre edge in the Voronoi diagram that partition the region of overlap between *C*_*i:j*_ and *C*_*i:k*_ is a great circle that passes through the two points that belong to all three spheres (shown as a dotted line). D) Cross section of the sphere *S*_*i*_ through the cutting plane indicated by two arrows in panel C. *C*_*i:j;k*_ corresponds to a spherical diangle; its surface area is computed as a function of the cap height, *h*_*i:j*_ , and the angle *θ* between the plane containing the great circle and the plane defining the cap *C*_*i:j*_ [4, 5].

The intersection between these two spheres is the union of two caps *C*_*i:j*_ and *C*_*j:i*_ these two caps are connected at the level of the plane that separates the Voronoi cells of *S*_*i*_ and *S*_*j*_. When the sphere *S*_*i*_ is in contact with more than one sphere, say with spheres *S*_*j*_ and *S*_*k*_, there is a possibility that the corresponding caps *C*_*i:j*_ and *C*_*j:i*_ overlap: this occurs when the triangle *Δc*_*i*_ *c*_*j*_ *c*_*k*_ is part of the dual complex *K*. Figure 3A illustrates this problem. To remove the ambiguity in assigning the corresponding overlap region *C*_*i:j;k*_ to either the contact between *S*_*i*_ and *S*_*j*_ or the contact between *S*_*i*_ and *S*_*k*_, we use the Laguerre Voronoi diagram on the surface of *S*_*i*_.

Sugihara [2] extended the concept of Laguerre diagram in the plane to a Laguerre Voronoi diagram on the surface of a sphere. In his approach, the Laguerre distance from a point *P* to a circle *C*_*i*_ on the sphere is defined as the geodesic length of the tangent line segment from the point to the circle. Similar to the Voronoi diagram described above, this distance function creates Laguerre Voronoi regions for each cap and separates them with geodesic arcs (see Figure 3B for an example of the Laguerre Voronoi diagram of ten circles on a sphere). We note that many of the properties of the weighted Voronoi diagram remain true in its spherical version. For example, if two circles intersect in two points, their Voronoi edge contains these two points.

The definition of contacts based on the alpha shape theory given above leads to the following additive property for all contact areas associated with a sphere *S*_*i*_:

$$4\pi r_{i}^{2}=A(S_{i})+\sum_{j}A_{i:j}$$

where *A(S*_*i*_ *)* is the surface area of *S*_*i*_ not covered by any other sphere, *A*_*i:j*_ is the contact area between the spheres *S*_*i*_ and *S*_*j*_, and the summation extends to all spheres *S*_*j*_ such that ***c***_***i***_ ***c***_***j***_ is an edge in the dual complex *K*.

There is a trivial correspondence between the Laguerre Voronoi diagram of the caps on the surface of sphere *S*_*i*_ and the set of simplices in the dual complex *K* that are associated with *S*_*i*_. For example, the two caps *C*_*i:j*_ and *C*_*i:k*_ overlap and share an edge in the spherical diagram if and only if the simplex *s*_*ijk*_ corresponding to the triangle formed by the centers of the three spheres *S*_*i*_, *S*_*j*_, and *S*_*k*_, belongs to *K*. This leads to the following inclusion-exclusion formula for the contact areas between a sphere *S*_*i*_ and its neighbors:

$$A_{i:j}=A(C_{i:j})-\sum_{k|S_{ijk}\in K}A\left(C_{i;j;k}\right)+\sum_{k,l|S_{ijkl}\in K}A\left(C_{i;j;kl}\right)$$

Here *A*(*C*_*i:j;k*_) is the area of the contribution of *C*_*i:k*_ to the intersection of *C*_*i:j*_ and *C*_*i:k*_, and *A*(*C*_*i:j;kl*_) is the common contribution of *C*_*i:k*_ and *C*_*i:l*_ to the intersection of *C*_*i:j*_ and *C*_*i:k*_. The computations of the different types of terms on the right side of this equation involve simple spherical geometry [5]. In Figure 3D, we illustrate the computation of *A*(*C*_*i:j;k*_).

**Figure 4:**
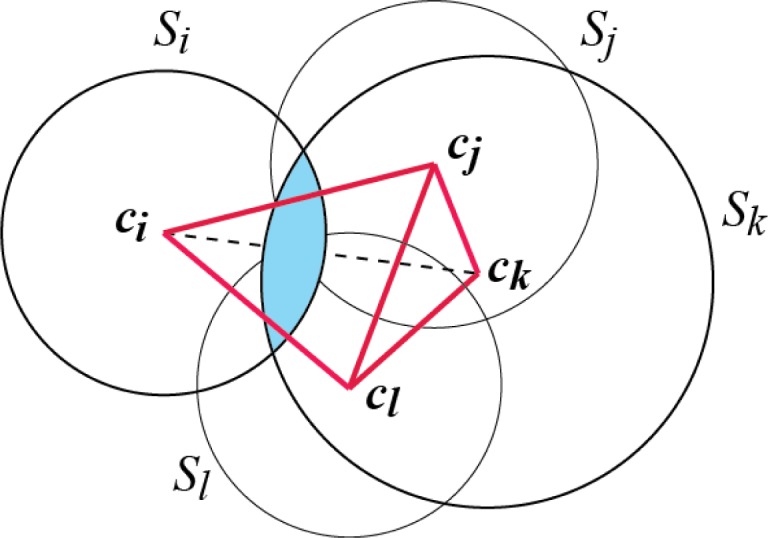
**Difference between sphere overlap and sphere contact**. Let us consider for illustration four circles *S*_*i*_, *S*_*j*_, *S*_*k*_, and *S*_*l*_ in the plane. Their dual complex, shown with solid red lines, is the union of the two triangles Δ*c*_*i*_ *c*_*j*_ *c*_*l*_ and Δ*c*_*j*_ *c*_*k*_ *c*_*l*_ and all their sub-simplices. The edge *c*_*i*_ *c*_*k*_ (shown as a dashed line) is not part of this dual complex and therefore the circles *S*_*i*_ and *S*_*k*_ are not in contact, according to our definition based on the alpha shape theory. However, they do overlap, with the common intersection shown in light blue.

We note that the definition of contacts between spheres given here is different from the standard definition based on local geometric proximity. Indeed, two spheres may overlap (i.e. be close in space) without being connected by an edge in *K* and therefore would not be considered in contact according to our definition (see Figure 4 for an illustration of this point). Our approach however is similar to the methods that define contacts in proteins based on the Voronoi diagram [88, 89, 92, 100].

#### Implementation

The theory described above provides a framework for measuring a union of spheres, i.e. computing its accessible surface area and enclosed volume, detecting its cavities and pockets, as well as for locating neighboring spheres in the union and defining their contacts. The implementation of this theory involves five steps: (i) compute the Delaunay triangulation, (ii) generate the dual complex, (iii) compute the surface area and volume using the Volume Theorem given above and the corresponding Area Theorem, (iv) detect pockets and cavities using the concept of flows described above, and (v) calculate individual contact areas using the contact definition described above. Several implementations of step (i) to (iv) are available, such as AlphaShape, CASTp [6, 101], and AlphaVol [7]. We have recently developed a new implementation of the same four steps that enables the analysis of very large molecular systems with millions of atoms, such as viral envelopes, available in the program UnionBall [96]. The addition of step (v) within the alpha shape theory is new and currently available in just one software package, BallContact [5].

## 5. Applications

The alpha shape theory provides an accurate and robust method for computing the geometric measures of a macromolecule. Among these measures, surface area and volume are used to quantify the interactions between such a molecule and the water surrounding it in implicit solvent models. The detection of pockets within a macromolecule and the determination of their sizes serve as a starting point for predictive studies of macromolecule-ligand interactions. In addition, the determination of internal atomic contacts allows for better characterization of atomic interaction and better definitions of solvation energies (see for example [102]. We provide illustrations of two of these applications of the alpha shape theory to study macromolecules, namely the characterization of pockets in ribosomes and the quantification of residue environment in protein structures. We then review recent applications of the alpha shape to study the geometry of large biomolecules and its relationship to function.

### 5.1 The geometry of ligand binding sites

**Figure 5:**
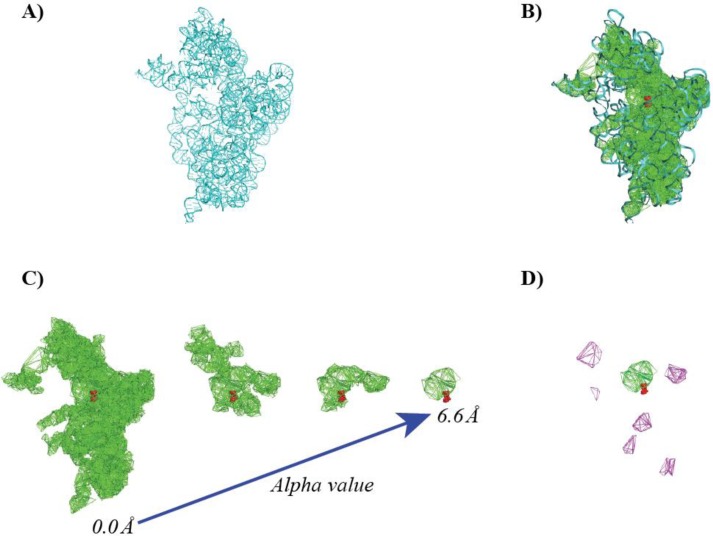
**Detecting pockets in the small ribosomal subunit 30S**. **(A)** The rRNA component of the 30S subunit of the ribosome of *Thermus thermophilus* (PDB code 1HNZ). **(B)** UnionBall detects a large pocket, shown in green that contains the binding site of hygromycin B, shown in red. **(C)** Evolution of the largest pocket found in the 30S ribosomal subunit, as we increase the parameter alpha from 0 Å to 6.6 Å. Note that the pocket remains in the vicinity of the binding site of hydromycin B. The RNA structure is omitted for sake of clarity. **(D)** Unfortunately, UnionBall does not distinguish between the pocket corresponding to the binding site of the antibiotic (pocket shown in green, and antibiotics in red), from other deep pockets that appear at large alpha value (shown in purple), although the green pocket is the largest.

The high-resolution structures of bacterial ribosomes, and those of their complexes with antibiotics, have significantly advanced our understanding of drug-RNA interactions, and paved the way for new antibacterial drug discovery and design, with the ribosome as a target. A prerequisite to drug design is the determination of the sites where the ligand may interact with its receptor. Binding sites of small molecule ligands are usually located in pockets (also referred to as clefts, or grooves) or cavities (i.e. pockets fully inaccessible to solvent) in the target macromolecule. As described in the previous section, the alpha shape theory provides the theoretical background that allows us to detect and measure these pockets. We have tested the performance of our own implementation, UnionBall [96], by checking if it is able to detect geometric pockets in the 30S subunit of the ribosome of *Thermus thermophilus* that are biologically relevant. The small ribosomal subunit is extensively studied as an antibiotic target, and there are at least eight structures of their complexes known [103]. We use the structure of the complex hygromycin B - 30S as a reference (PDB code 1HNZ). Figure 5 shows the results of the application of UnionBall on the 30S ribosome. Note that all calculations were performed in the absence of an antibiotic molecule. We found that the deepest pocket, i.e. the largest pocket identified with a large alpha value, matches with the position of the antibiotics that binds to the 30S subunit of the ribosome.

### 5.2 Residue environment in protein structures

It is common to characterize the structural environment of a residue in a protein from the secondary structure element it belongs to and its accessible surface area [104]. The former characterizes the local conformation of the residue, while the latter is used to quantify the surface area that was buried upon folding, as it is expected to differ for hydrophilic and hydrophobic residues. This has led to a quantification of the hydrophobic effect using the concept of a water-implicit solvation free energy that is computed as a weighted sum of the accessible surface areas of all residues in a protein [11]. We have extended this idea by accounting for the nature, and extent of, the inter-atomic contacts that are formed in the core of the protein as it folds [102, 105]. Here we show why the nature of the inter-atomic contacts matters.

The fraction of the surface area of any atom that is in contact with solvent is called the solvent accessible surface area (ASA). In parallel, we define the polar contact surface area, or PCA, and the non-polar contact area, or NPCA, of an atom as its area in contact with (or occluded by) polar and non-polar atoms, respectively. In all analyses presented below, carbon and sulfur atoms were classified as non-polar atoms, while nitrogen and oxygen (neutral or charged) were classified as polar atoms. Note that PCA and NPCA should not be confused with the polar surface area and non-polar surface area, which commonly correspond to the accessible surface area of polar and non-polar atoms, respectively. All surface areas mentioned above (i.e., ASA, PCA, and NPCA) were computed based on the alpha shape theory and its definition of contacts.

The calculation is performed with the program BallContact as follows. Each atom of the protein is represented as a ball, centered at the position of the atoms in the minimized structure for the protein, with a radius equal to *R*_*vdW*_ *+R*_*H2O*_, where *R*_*vdW*_ is the vdW parameter for the atom in AMBER94 and *R*_*H2O*_ is the radius of the solvent probe, set to 1.4 Å. For an atom *i* in the protein, the program outputs its accessible surface area, ASA, as well as the list of atoms that are in contact and the corresponding contact areas. These atoms are then divided into two groups, those that are “near” (following the terminology of Shrake and Rupley [46]), i.e. that belongs to the same residue as *i* or to the backbone of the two flanking residues, and the others, named “long”. Atoms that are “near” account for the stereochemistry of the residue to which atom *i* belongs and are not included in the subsequent calculations. Contact atoms that are “long” are further subdivided into polar and non-polar atoms, according to the definition above; the PCA and NPCA surface areas are then the sum of the corresponding contact areas.

We define the environment of a residue in a protein as the union of the accessible areas of its atom and of all their “long” contacts. This environment is then divided into an ASA, PCA, and NPCA. These three values correspond to sums of areas on spheres, given in Å^2^; they are independent of each other. We define corresponding normalized values, XASA, XPCA, and XNPCA, according to:

$$XASA=\frac{ASA}{SAS+PCA+NPCA};\;XPCA=\frac{PCA}{SAS+PCA+NPCA};\;XNPCA=\frac{NPCA}{SAS+PCA+NPCA}$$

These three fractions of surface areas, expressed in percent, are no longer independent, as their sum is 100.

**Figure 6:**
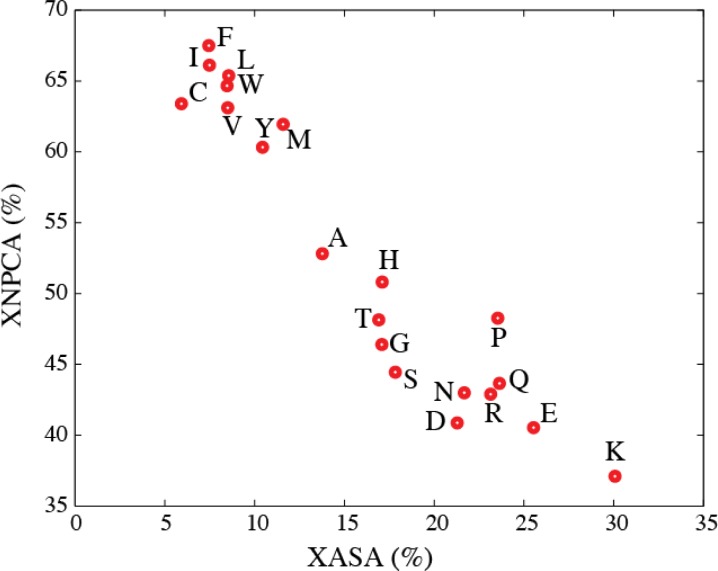
The mean residue non-polar environment (XNPCA) is plotted against the mean residue solvent environment (XASA) for all twenty types of amino acids; XNPCA and XASA were computed from a set of 305604 residues from a database of 1555 high-resolution protein structures.

We collected data on the environments (accessible to solvent, polar, or non-polar) of 305604 residues in a database of high-resolution protein structures [5]. The corresponding average results are shown in Figure 6.

We found that the non-polar environments of all twenty types of amino acids are weakly correlated to their accessible environments. This weak correlation illustrates that accessible surface area and contact areas provide complementary information that is relevant to the native conformations of the proteins. We note that the plot of non-polar contact area versus accessible surface area partitions the amino acids into two groups, those with low ASA and high NPCA, namely C, V, I, L, M, Y, F, W, and the others; this partitioning parallels the groupings of amino acids as being either hydrophobic or hydrophilic.

### 5.3 Recent applications of the alpha shape theory to study macromolecules

The alpha shape theory was originally developed in the early 1990s by Edelsbrunner and co-workers to characterize the shapes of sets of points (weighted or not) in 2D and 3D [67, 106, 107]. As weighted points can be seen as balls, and as molecules are usually represented as union of balls, it was not surprising to see alpha shapes being adapted to characterize the shapes of molecules. The first applications focused on measuring molecular shapes (i.e. computing their volume and surface area) [95] as well as on characterizing the “empty spaces” enclosed within the boundary of a molecule, namely cavities [84] and pockets [6, 85]. While these applications of the alpha shape theory remain popular in structural biology with new and improved software implementations being released regularly, such as AlphaVol [7], CASTp [101], Vorlume [108], and UnionBall [96], many applications in new domains have been proposed. Here we review a few of these applications.

#### Statistics of protein structure geometry

Proteins are essential tools that perform a wide variety of biological functions inside the cell. Just like in the case of macroscopic tools, it is the shape and dynamics of a protein that define its function. Recent structural genomics initiatives have undertaken the vast challenge of finding the structures of all known proteins, in hopes of unraveling this relationship between geometry and function. The experimental determination of a protein structure at the atomic level remains, however, a difficult problem. There is hope however that theoretical and computational techniques will supplement experimental methods and enable protein structure prediction at the near atomic level [109, 110]. Many of these techniques rely on the knowledge derived from the analysis of the geometry of known protein structures. Such an analysis requires an objective definition of atomic packing within a molecular structure. The alpha shape theory has proved a useful approach for deriving such a definition. Singh et al for example used the Delaunay complex to define nearest-neighbors in protein structures and to derive a four-body statistical potential [111]. This potential has been used successfully for fold recognition, decoy structure determination, mutant analysis, and other studies (for a full review, see [112]). The potentials considered in these studies rely on the tetrahedra defined by the Delaunay triangulation of the points representing the atoms. In parallel, Zomorodian and colleagues have shown that it is possible to use the alpha shape theory to filter the list of pairwise interactions to generate a much smaller subset of pairs that retains most of the structural information contained in a proteins [113]. The alpha shape theory has also been used to characterize the shapes [114] and surfaces [115–117].

#### Protein structure alignment

The alpha shape theory allows for the detection of independent simplices characterizing the geometry of a protein structure. It is worth mentioning that it is possible to use this information to compare two protein structures and even to derive a structural alignment between these structures [118, 119].

#### Characterizing and predicting bio-molecular interactions

As the function of a protein is related to its geometry and as function usually involves binding to a partner protein, significant efforts have been put into charactering the geometry of protein-ligand interactions, where ligands include small molecules, nucleic acids, and other proteins. Among these efforts, a few relate to the applications of the alpha shape theory. As described for example in Figure 5, the latter has been used extensively for detecting pockets and cavities within molecules that are putative binding sites [6, 101]. It has been recently extended to characterize binding sites at the surface of proteins [115–117, 120, 121]. The alpha shape theory has also been used to characterize the interfaces in protein-protein complexes [122] as well as protein-DNA interactions [123]. For a complete review of the applications of the alpha shape theory to characterize protein interactions, the reader is referred to [124].

It is worth mentioning a geometric parallel between finding a structural alignment between two proteins and predicting the structure of their interactions. While the former is based on the identification of similar geometric patterns between the two structures, the latter is based on the identification of complementary patterns between the surfaces of the two structures. As mentioned above, geometric patterns based on the Delaunay triangulation have been used for structural alignment. In parallel, similar patterns have recently been used to predict protein-protein interactions [125].

#### Alpha shapes as a tool to characterize dynamics

All the applications described above relate to the static geometry of molecules. Bio-molecules however are dynamics. A molecular dynamics simulation is designed to capture this dynamics: it follows the Newtonian dynamics of the molecule as a function of time, generating millions of snapshots over the course of the trajectory [126]. The alpha shape theory has proved useful to characterize the geometric changes that occur during such a trajectory. For example, using the concept of topological persistence [127], Kasson et al characterized structural changes in membrane fusion over the course of a simulation [128]. More recently, Lindow et al proposed a a Voronoi-based algorithm to extract the geometry and the dynamics of cavities and channels from a molecular dynamics trajectory [129].

## 6. Summary and Outlook

The Alpha Shape Theory provides a fast, accurate, and robust method for characterizing the geometry of a macromolecule represented as a union of balls. In this paper, we have presented the mathematical foundations of this theory and described its applications to measuring the shape of a molecule. We have shown how it can be used to compute the volume and surface area of a union of balls, to detect and measure cavities and pockets inside the outer envelope of such a union of balls, and to compute the surface areas of the contacts between the balls. We have reviewed how these measures are related to properties of the molecule of interest, as well as recent applications of the alpha shape theory that go beyond studying the geometry of a single molecule. We conclude this paper with a description of one new challenge in biology in which the alpha shape theory is expected to prove useful.

Recent advances in structural biology have produced an abundance of data on large macro-molecular complexes such as the RNA polymerase transcription complexes, the ribosome complexes, as well as large viral particles with more than sixteen million atoms. Modeling the dynamics of such large systems is as important as modeling smaller proteins. It becomes impractical, however, to consider all atoms of such molecular machinery and we need to introduce approximations that consider the system at coarser levels of detail. One possible approach is to represent the macro-molecular complex with a small number of spheres, supplemented with a model for their interactions that captures the physics of the underlying atomic model. This model will include a potential energy function for internal interactions and a potential energy function to account for the solvent environment of the system. We expect the latter to resemble the solvation potentials described in these papers that relate geometry and energy. We also expect the alpha shape theory, which provides full characterization of union of balls or spheres, to play an important role in both characterizing the coarse-grained representations of these molecular machines and in developing the models for their interactions.
